# Interaction of Huanglongbing and Foliar Applications of Copper on Water Relations of *Citrus sinensis* cv. Valencia

**DOI:** 10.3390/plants8090298

**Published:** 2019-08-23

**Authors:** Said A. Hamido, Robert C. Ebel, Kelly T. Morgan

**Affiliations:** Southwest Florida Research and Education Center, University of Florida, 2685 SR 29 N, Immokalee, FL 34142, USA

**Keywords:** greening, *Candidatus* Liberibacter asiaticus, *Xanthomonas citri* subsp. *citri*, citrus canker, essential nutrients, stem water potential, stomatal conductance

## Abstract

The following study was conducted to determine the impact of frequent foliar Cu applications on water relations of Huanglongbing (HLB)-affected *Citrus sinensis* cv. ‘Valencia’. HLB in Florida is putatively caused by *Candidatus* Liberibacter asiaticus that is vectored by the Asian citrus psyllid. The experiment was conducted in a psyllid-free greenhouse with trees grown in Immokalee fine sand soil with the trees well-maintained to promote health. Cu was applied to the foliage at 0×, 0.5×, 1×, and 2× the commercially recommended rates, which were 0, 46, 92, and 184 mM, respectively, with applications made 3× in both 2016 and 2017. Previous studies indicate that HLB causes roots to decline before the canopy develops symptoms, which increases the ratio between the evaporative surface area of the canopy to the uptake surface area of roots and increases the hydraulic strain within the tree. In the current study, overall growth was suppressed substantially by HLB and Cu treatments but the ratio between evaporative surface area (leaf surface area) and the uptake surface area of roots (feeder root surface area) was not affected by either treatment. Stem water potential (Ψ_xylem_), which was used as a measure of plant water deficits and the hydraulic strain within the tree, was significantly 13% lower for HLB-affected trees than the non-HLB controls but were not affected by Cu treatments. All Ψ_xylem_ measurements were in a range typical of well-watered trees conditions. Stomatal conductance (k_s_) and root and soil resistances (R_r+s_) were not affected by HLB and Cu. The results of this experiment suggest that tree leaf area and feeder roots are reduced when the trees are affected by HLB or are treated with foliar Cu applications such that plant water deficits are not significantly different over that of the controls.

## 1. Introduction

Huanglongbing (HLB) in Florida is a disease putatively caused by the gram-negative bacteria *Candidatus* Liberibacter asiaticus (*C*Las) and vectored by the Asian citrus psyllid. HLB affects many aspects of citrus physiology that increase the plant’s susceptibility to secondary stress and which collectively promote whole-plant decline [1]. HLB is compelling commercial citrus grove managers to extensively alter their management practices. One of the earliest and the most important symptoms that have been reported for HLB is debilitation of the root system, especially feeder (fibrous) roots [2]. 

Since feeder roots are responsible for 90% of water uptake [3,4], their debilitation in HLB-affected trees has been implicated as the primary cause of higher plant water deficits, as measured by lower stem water potential (Ψ_stem_), in a controlled greenhouse study [5]. More recent evidence indicates that HLB also thickens xylem cell walls that reduce lumens of xylem vessels [6] that may affect the resistance of water movement through the tree, and thereby, impact plant water deficits. 

HLB causes citrus trees to differentially regulate genes, which leads to catabolism of their own cell walls [1] and how this may affect water transport in xylem is unknown. The higher plant water deficits of HLB-affected trees have been shown to be better alleviated by high-frequency irrigation than the less frequent irrigation that has been traditionally recommended [7]. The authors demonstrated that under Florida climate conditions, where there are distinct “rainy” and “dry” periods, high-frequency irrigation was shown to be most critical when trees were actively growing during the spring “dry” season (February–May), which coincides with flowering, fruit set, and early growth flushes [7]. Even during the “rainy” season (June–September), which is the period that encompasses most of fruit enlargement, high-frequency irrigation alleviated internal plant water deficits, as measured by Ψ_xylem_ [7].

Multiple sprays of Cu throughout the growing season are the most widely used bactericide treatments in commercial citrus groves in Florida to suppress *Xanthomonas citri* subsp. *citri* (*Xcc*), the causal agent of citrus canker, and were used previously to suppress fungal pathogens [8]. Because there is no other economically viable alternative to control canker disease in citrus, in 2017, growers sprayed ≈ 408 Metric tons of copper hydroxide (Cu (OH)_2_) on ≈ 149 thousand ha of orange trees in Florida [9]. Frequent sprays promote toxic levels of Cu in soils that suppress root growth of trees [10,11,12]. Unfortunately, it is not fully understood how HLB-affected citrus trees respond to excess Cu.

In the first part of the research in this series, we demonstrated that HLB-affected trees are more sensitive to foliar applications of Cu than non-HLB trees [13]. Cu was applied to the foliage and a few days later, excess Cu was washed off leaves and onto the soil surface where it moved into the soil profile. HLB-affected roots acidified soil more than non-HLB controls making soil Cu more readily available, which in turn, promoted greater accumulation of Cu in their roots and leaves. The higher Cu contents of HLB-affected trees suppressed overall growth more than the non-HLB controls. 

The purpose of the current study was to determine how HLB and frequent foliar applications of Cu impact water relations of citrus and which parameters are responsible for any changes. With debilitation of root systems occurring before the canopy demonstrates symptoms for HLB-affected citrus trees, it is reasonable to hypothesize that this phenomenon increases plant water deficits that suppress plant growth. 

Thus, the objectives were to determine how foliar Cu applications for disease control influence water relations of HLB-affected citrus trees.

## 2. Materials and Methods

### 2.1. Plant Culture and Treatments

The current experiment was conducted in a psyllid-free greenhouse at the University of Florida, Southwest Florida Research and Education Center (SWFREC) near Immokalee, Florida, USA (lat. 26.42° N, long. 81.42° W) from 2016 to 2017. The trees used were seven-year-old *Citrus sinensis* (L.) Osbeck cv ‘Valencia’ on Swingle citrumelo (*Citrus paradisi x Poncirus trifoliata*) rootstock. One-year-old trees were obtained from a commercial nursery in 2009, repotted into 10 L pots, and double budded with buds highly infected with *Candidatus* Liberibacter asiaticus (*C*Las), as reported previously [14]. The trees had been well-maintained in the psyllid-free greenhouse with daily watering, commercial recommendations of fertilization, and suppression of insect pests before the current study was initiated. In April 2016, 48 trees (24 with HLB and 24 non-HLB controls) were transplanted into 100 L pots with the potting media consisting of Immokalee fine sand soil (Sandy, siliceous, hyperthermic Arenic Alaquods). The trees were allowed to become established for three months and were approximately 1.5 m in height before Cu treatments were initiated. Fruit were removed at the start of the experiment and after fruit set in 2017. 

All trees used in this experiment were tested for the presence of *C*Las, and HLB-affected trees were confirmed on 15 February 2016 by RT-PCR [15]. The cycle time (Ct) of HLB-affected trees averaged 24.9, which was below the threshold of 32. The trees exhibited very mild HLB symptoms, including earlier growth and bloom in spring, delayed greening of newly developing leaves with veinal chlorosis, interveinal chlorosis, and whole leaf chlorosis present, retarded leaf and shoot growth, and reduced growth. The HLB-affected trees were, on average, about half the size of the non-HLB controls, however, there was wide variation in tree size that allowed selecting trees based on total plant leaf area such that the average leaf area/tree was similar for HLB-affected and control trees and across Cu treatments. This approach removed bias in tree size across all treatments when the experiment was initiated. Total leaf area was determined by counting leaves and measuring 10 leaves per tree, as described by Ebel et al. [13].

The trees were watered daily using microjets that wetted most of the soil surface until water dripped from the bottom of the pots. The trees were fertilized with 20-2-20 NPK with 35 g/tree contained 0.74%, 1.1%, 0.1%, 0.05%, 0.05%, 0.025%, and 0.025% of S, Mg, Fe, Mn, Zn, Cu, and B, respectively, (Peat-Lite Low Phos Special, Peters Professional, Allentown, PA, USA), every two to five weeks from July 2016 through August 2017. Each tree received an annual amount of 152, 15, and 152 g N, P, and K, respectively. The trees were also given a foliar application containing 0.3%, 1.2%, 3.6%, 5.0%, 1.1%, 0.003%, and 2.7% of P, Ca, Mg, Fe, Mn, Zn, Cu, and B, respectively, (special formulation, Peters Professional, Allentown, PA, USA) at 0.8 and 2.1 g/tree on 19 October 2016 and 1 November 2016, respectively. 

Cu treatment simulations were conducted by applying 0, 0.5×, 1.0×, and 2.0× of the commercially recommended rate to trees on 19 July, 11 August, and 30 August in 2016 and 2 May, 6 June, and 26 July in 2017. The rates equaled 0, 46, 92, and 184 mM, respectively, using Cu (OH)_2_ (Kocide 2000, I.E. DuPont Canada Co., Mississauga, ON, Canada) with 2.5 L of solution applied/tree. Four days after treatment, the canopy of each tree was rinsed with 2 L water to remove residual copper from the foliage and move it onto the soil surface. This procedure somewhat simulated commercial conditions that exist during the rainy season in Florida, although under natural conditions precipitation is usually more frequent and more lengthy than what was practiced in this study. 

### 2.2. Foliar Cu Analysis

Leaf samples were collected to determine nutrient content using the procedures of Obreza and Morgan [16] and processed according to tissue analytical methods [17,18,19]. Leaf samples of 10 mature, fully expanded leaves were randomly collected from each tree and rinsed to remove residues in 0.2 M HCl and dried for 72 h at 60 °C. Once the tissues reached a constant weight, they were ground in a mill until all tissue could pass through a 60-mesh sieve and mixed thoroughly [13]. Tissue Cu concentrations were determined using a dry ash combustion digestion method [20]. A 0.5 g sample of dried ground leaf material was weighed and dry ashed at 500 °C for 16 h [17]. The ash was equilibrated with 15 mL of 0.5 M HCl at room temperature for 0.5 h. The solution was drained into 15 mL plastic disposable tubes and kept in a refrigerator at ≤4 °C [19] until analyses by inductively coupled plasma atomic emission spectrometry (ICP-AES; OPTIMA 7000DV, Perkin-Elmer, Billerica, MA, USA) according to Munter and Grande [21]. Tissue nutrient concentration was compared with critical levels for Florida citrus [16,22]. 

### 2.3. Theoretical Model and Measurements to Aid Interpretation of the Impact of HLB and Cu Treatments on Plant Water Relations

All measurements involving water relations of the trees in the current study were conducted during the steady-state conditions that occur at midday (noon–6 p.m.).

Steady-state conditions are defined by transpiration of the entire plant canopy being equal to water uptake by roots, which can be expressed as:

T_p_ = U_p_ (1)

where T_p_ = whole plant transpiration (mmoles·s^−1^) and U_p_ = uptake of water by the root system (mmoles·s^−1^).

Stomata serve as the most important regulator of plant water deficits and partially close at midday to prevent excessive hydraulic strain within the plant. Regulation of stomatal aperture has been a major area of study for decades, where it is generally accepted that they are regulated by plant water deficits through signals that are produced within the plant [23]. We can express the impact of stomatal aperture on water flow through the plant by:

T_p_ = [(*ρ*_vs_ − *ρ*_va_)/(r_vs_ + r_va_)]T_sa_ (2)

where T_p_ is the whole plant transpiration (mmoles·s^−1^), *ρ*_vs_ − *ρ*_va_ (mmoles·m^−3^) is the vapor pressure deficit between the substomatal cavity (*ρ*_vs_) and the outer atmosphere (*ρ*_va_), r_vs_ (stomatal resistance) + r_va_ (aerial resistance) (s·m^−1^), and T_sa_ (m^2^) is the surface area of the canopy involved in transpiration, which is required to be included in the model for the units to balance on both sides of the equation. The resistances include the resistance of water movement through the stomatal pore (r_vs_) and the boundary layer resistance (r_va_) that extends from the stomatal pore to the outer atmosphere and which are cumulative since they are in series. Stomatal conductance (k_s_), which is the measurement that is typically made in plant physiological studies, is the reciprocal of r_vs_ (k_s_ = 1/r_vs_). 

#### 2.3.1. T_p_ (mmoles·s^−1^)

Whole plant transpiration was measured using weighing lysimeters (Locher Environmental Technology, Punta Gorda, FL, USA) attached to electronic data loggers (model CR10, Campbell Scientific, Logan, UT, USA). Plant weights were recorded at noon, which was two hours after irrigation so that gravitational water had percolated through the pot, and again at 6 pm to determine the weight change over the 6-h period. The T_p_ measurement included evaporation from the soil surface, but since the soil surface is constant across all treatments, transpiration measured using this approach still allows for statistical comparisons among treatments. Furthermore, evaporation from the soil surface is a minor component (<20%) of overall evapotranspiration [24].

#### 2.3.2. Stomatal Conductance (mmoles·m^−2^·s^−1^)

Stomatal conductance (k_s_) was measured in June, September, and December, 2016 and April and August, 2017 using a steady-state porometer (Model 1600, LI-COR, Inc., Lincoln, NE, USA). Measurements were taken between 12:00 and 3:00 p.m. on three leaves per tree with leaves perpendicular and exposed to the sun. Since units for r_vs_ in Equation (2) are s·m^−1^, the units for k_s_ are m·s^−1^, which can also be considered on a volumetric basis per unit area (m^3^·m^−2^·s^−1^), that is, a volume of water transpiring per unit leaf area per second. The porometer gives units in mmoles·m^−2^·s^−1^. Under the environmental conditions of this study, vapor density (mmoles·m^−3^) was relatively constant and thus the k_s_ units using the porometer do not introduce a significant error.

#### 2.3.3. T_sa_ (m^2^)

Most transpiration of citrus canopies occurs through stomates on leaves and since they are on the abaxial surface, the total plant leaf area can serve as T_sa_. However, T_sa_, as measured here, is an overestimation of true T_sa_, which is the total surface area of the stomatal pores. Total leaf area was determined every two to four weeks, as described by Ebel et al. [13].

Water uptake by roots is driven by the water potential gradient between the xylem and soil with the rate of uptake modified by the resistances along the path of flow:

U_p_ = (Ψ_soil_ − Ψ_xylem_)/R_r+s_ (3)

where Ψ_xylem_ is the water potential of the xylem (MPa), Ψ_soil_ is the water potential of the soil (MPa), and R_r+s_ is the root and soil resistances (MPa·mmoles^−1^·s), which are in series and, therefore, cumulative. Ψ_xylem_ is the most commonly used measure of plant water deficits for tree fruit crops as a direct measure of hydraulic strain within the plant and is relatively uniform throughout the canopy and varying by only 0.01 MPa·m^−1^ in elevation due to gravitational forces [4,5,7,25,26,27], and (3) for the simplicity in its measurement. R_r+s_ includes the resistance that water encounters as it traverses the soil to the root surface (R_s_) and the resistance encountered by the water having to traverse the endodermis (R_r_). 

A caveat to Equation (3) that must be considered is that the ratio between the transpiration surface area of the canopy and the uptake surface area of the root system (T_sa_/U_sa_) is directly related to the hydraulic strain within the plant and that without its inclusion in Equation (3), its impact is incorporated into the calculation of R_r+s_ [3,4]. The debilitation of roots by HLB and excess soil Cu should, at least temporarily, increase the T_sa_/U_sa_ ratio, and thus, remove its influence from the determination of R_r+s_ we can modify Equation (3):

U_p_ = (T_sa_/U_sa_)[(Ψ_soil_ − Ψ_xylem_)/R_r+s_] (4)

Equation (4) can then be rearranged to calculate R_r+s_:

R_r+s_ = (T_sa_/U_sa_)(Ψ_soil_ − Ψ_xylem_)/U_p_ (5)

#### 2.3.4. Uptake Surface Area of the Roots (U_sa_) (m^2^)

The majority of water uptake (>90%) occurs through feeder roots [3,4], however, measurement of the total surface area of feeder roots is not possible with roots growing in an opaque soil as in the current study. U_sa_ can be estimated by measuring the fraction of the feeder root surface area using clear rhizotron tubes and extrapolating that quantity to the whole pot. 

Clear rhizotron access tubes (52 × 6.4 cm) were placed into a hole augured vertically (90°) into the soil to the bottom of the pot at 15 cm away from the trunk and at 40 cm from irrigation emitters. Images were captured using a digital camera (model CI-600 In-Situ Root Imager, CID-Bioscience, Camas, WA, USA) that rotated with 360° and scanned the soil area within 21.59 × 19.56 cm layers. Roots were classified and examined using digital imaging software (Root Snap CI-690, version 1.3.2.25, CID-Bioscience, Camas, WA, USA). The scanner was calibrated according to the manufacturer’s instructions prior to sampling on each sampling date. The software segregated roots in the images by size at 1 mm increments with feeder roots considered to be ≤2 mm and structural roots ˃2 mm [6]. U_sa_ was estimated by finding the surface area of feeder roots measured through the rhizotron tubes and extrapolating the volume of the rhizotron tubes to the volume of the pots. 

#### 2.3.5. Feeder Root Lifespan

Another approach to determining the impact of HLB and Cu treatments on roots was to determine their lifespan. The lifespan of feeder roots was determined by selecting four feeder roots per tree from the rhizotron images and finding the number of days between the dates they emerged and disappeared. 

*Ψ_soil_ (MPa).* The trees were irrigated daily with irrigation being terminated by 10 am such that Ψ_soil_ was assumed to be zero.

*Ψ_xylem_ (MPa).* Xylem water potential of the stem (Ψ_xylem_) was determined in June, September, and December in 2016, and April and August in 2017 using a pressure chamber [28]. Two mature leaves per tree were randomly selected and covered with flexible plastic and aluminum foil 22 h prior to measurements. The leaves were severed at the petiole near the stem using a sharp razor blade and pressurized. Measurements were performed between 12:00 and 2:00 p.m.

*U_p_ (mmoles·s*^−1^*)*. Since T_p_ = U_p_, as expressed in Equation (1), measurement of T_p_, as described previously, serves as a measure of U_p_. 

### 2.4. Experimental Design and Statistical Analysis

This study was conducted as a 2 HLB treatment (HLB and non-HLB control) × 4 Cu application treatment (0×, 0.5×, 1×, and 2×, which correspond to 0, 46, 92, and 184 mM, respectively) factorial, completely randomized design. There were six replications per treatment, although some data were collected on only three reps per tree. All data were analyzed using the Statistical Analysis System (SAS for Windows, Ver. 9.4, SAS Institute Inc., Cary, NC, USA). Data analyzed over time (MAFT: Months after first Cu treatment) were analyzed using the Proc MIXED with time (month) included as a repeated measure. Means were separated by determining P > F of the least-square means (pdiff). Where data could not be analyzed over time, data were analyzed using Proc GLM and means separated using LSD. In addition, root lifespan/survival was analyzed using Kaplan–Meier survival analysis with SigmPlot (SigmaPlot 13, Systat Software, Inc., San Jose, CA, USA).

## 3. Results and Discussion

### 3.1. Foliar Cu Content 

**Figure 1:**
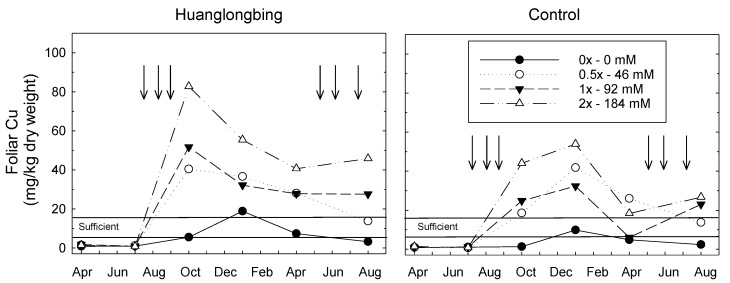
Interaction of HLB and foliar applications of Cu at 0×, 0.5×, 1×, and 2× the recommended rates (0, 46, 92, 184 mM, respectively) on foliar content of Cu. Vertical arrows indicate dates of Cu foliar treatments. Horizontal lines indicate the range that Cu is considered sufficient in citrus leaves [16].

The foliar content of Cu was low when the experiment was initiated, but leaves responded strongly to the foliar applications of Cu (Figure 1). There was a significant HLB*Cu*Months after first Cu treatment (MAFT) interaction (P > F <0.01) with foliar Cu higher for HLB-treated plants and higher with increased Cu applied (Table 1). Foliar Cu increased over time, however, the difference between HLB treatments can be most simply understood by considering the main effect means, which were 17.1 mg·kg^−1^ for HLB-affected leaves and only 12.0 mg·kg^−1^ for the non-HLB control leaves. As expected, foliar contents were higher with increasing Cu application concentrations with the average Cu content of controls 4.9 mg·kg^−1^ dry weight whereas the leaves that received 184 mM Cu had 23.7 mg·kg^−1^ dry weight, representing a ≈ 5× increase. The first paper in this series demonstrated that the pH of the soil in which HLB-affected trees grew was lowered compared with the soil in which non-HLB controls were grown. Reduced soil pH solubilized soil Cu and promoted higher uptake in HLB-affected trees than the non-HLB control [13]. The low pH-induced solubilization of soil cations has a reason for nutrient uptake and improved fertilization management of commercial citrus groves with HLB.

### 3.2. Leaf Area (T_sa_)

**Figure 2:**
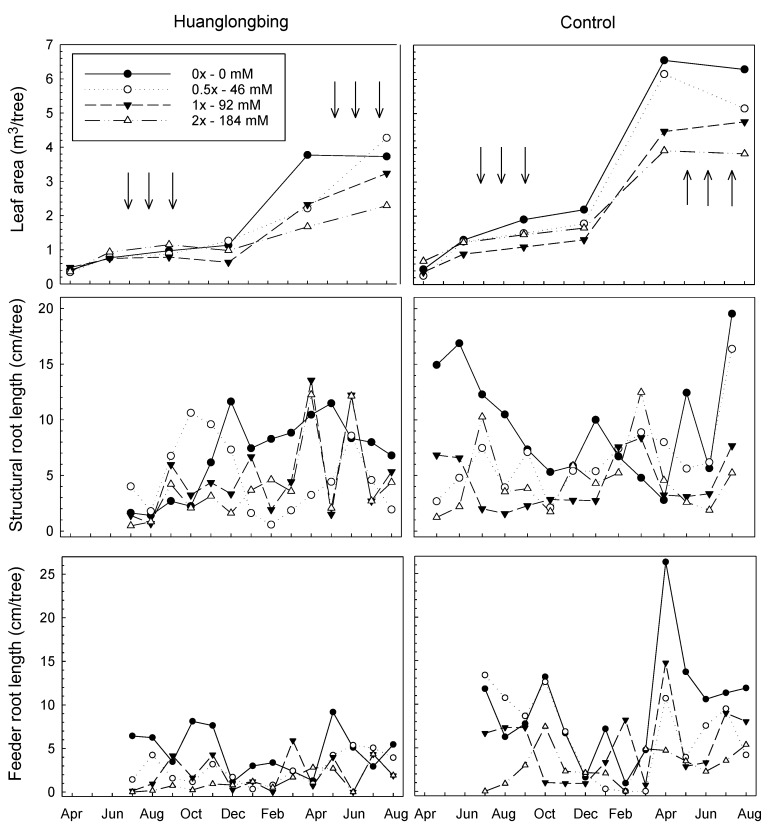
Interaction of HLB and foliar applications of Cu at 0×, 0.5×, 1×, and 2× the recommended rates (0, 46, 92, 184 mM, respectively) on leaf area, observable structural and feeder root lengths of *Citrus sinensis* cv. ‘Valencia’. Vertical arrows indicate the dates of Cu foliar treatments. Four days after each foliar application of Cu, the tree leaves were rinsed with water to mimic the field conditions.

Transplanting the trees into pots that were 10× larger than before the experiment created a rooting environment that encouraged strong canopy growth, as shown by the increase in T_sa_ (Figure 2). T_sa_ was reduced for HLB-affected trees compared to the non-HLB control trees and was reduced with increasing Cu concentrations of the foliar treatments as indicated by the significant HLB*Cu*MAFT interaction (P > F = 0.03). The HLB-affected trees had, on average, about half the leaf area (2.1 m^2^) of the non-HLB control trees (3.4 m^2^). As a result, the average daily increase of leaf area was estimated to be 49 and 110 cm^2^ day^−1^ under HLB-affected and healthy trees, respectively. Measured leaf area at the end of the experiment was 8.1 and 14.9 times greater than the beginning of the experiment for HLB-affected and healthy trees, respectively (data not shown). These data indicate the negative effect of HLB on citrus leaf area developments. Furthermore, increasing Cu rate significantly reduced the leaf area development under both HLB-affected and healthy trees. Higher Cu rate significantly reduced the leaf area growth by 52% and 30% for HLB-affected and healthy trees, respectively. Average monthly leaf area increase was from 0.13 to 0.29 m^2^ under HLB-affected and healthy trees, respectively. These data elucidate the impact of the higher Cu rate on reducing citrus trees leaf area which could be equal to the effect of HLB on tree leaf area development. Measured leaf area at the end of the experiment under higher Cu rate was 6.74 and 8.86 times greater than the beginning of the experiment for HLB-affected and healthy trees, respectively. Similar observations were reported by various researchers under different crops and all of them indicated a suppression effect of increasing Cu rate on plant development [29,30,31,32]. For example, MacFarlane and Burchett [31] indicated that increasing Cu rate significantly reduced tree leaf number and leaf area. 

### 3.3. Total Observable Root Length, Uptake Surface Area (U_sa_), and Feeder Root Lifespan

Total observable structural and feeder root lengths were impacted by HLB and the Cu treatments, as shown by the HLB*Cu treatment*MAFT interactions (P > F < 0.01), but the amount of structural and feeder roots varied widely throughout the course of the study (Figure 2). The variation in root growth over time is typical of plants, being affected by such factors such as the stage in growth flush [33] and fertilization [34]. The lengths of structural (4.0 cm) and feeder (2.5 cm) roots were reduced by about half for HLB-affected roots compared to the non-HLB controls (7.7 and 5.3 cm, respectively) and both root types declined to about 1/3 of the controls (9.5 and 7.1 cm, respectively) for the highest Cu treatment (3.4 and 2.1 cm, respectively) as indicated by the main effect means. Thus, the study clearly demonstrated that increasing Cu rate had a significantly greater detrimental impact than HLB on root growth. However, average root length growth (the total root increase divided by the period of the experiment) was estimated to be 3.2 or 4.2 mm day^−1^ in HLB-affected trees and healthy trees, respectively. Rhizotron methodology has been widely used to study the root dynamics in seasonal crops [35,36] and perennial trees [33,37,38,39,40,41,42]. A few studies have reported a total root length measurement of different trees with an estimated average between 3 and 5 mm day^−1^ [43,44,45]. Our results for healthy trees were similar to those reported by Bevington and Castle [11] where the root length of Carrizo citrange increased by ≈10 mm day^−1^ during early spring. 

The analysis for U_sa_ is the same as that for total root length of the feeder roots since the determination of U_sa_ is based on the multiplication of the total feeder root length by a constant (analysis not shown).

**Figure 3:**
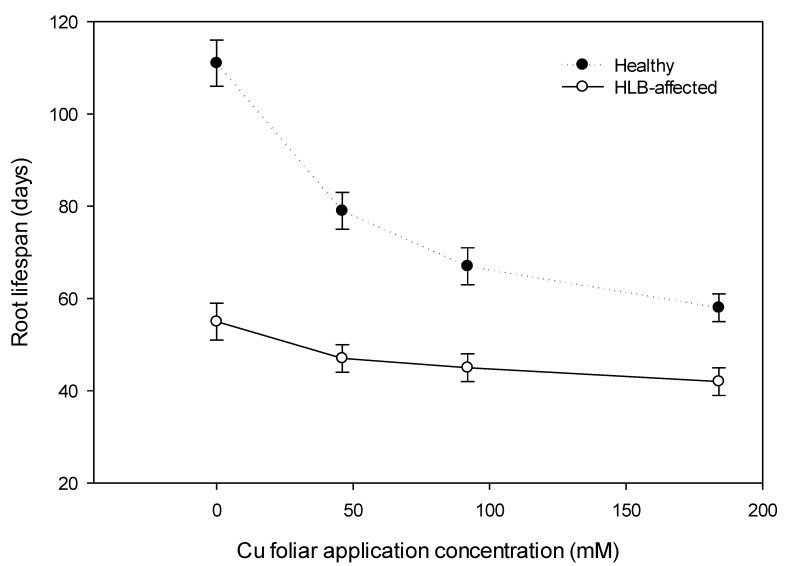
Interaction of HLB and foliar applications of Cu at 0×, 0.5×, 1×, and 2× the recommended rates (0, 46, 92, 184 mM, respectively) on feeder root lifespan of *Citrus sinensis* cv. Valencia. Bars represent the standard errors of the means.

Consistent with the suppression in total feeder root length by HLB and Cu treatments, root lifespans were shortened by the same treatments. There was a significant HLB * Cu treatment on the lifespan of roots (P > F < 0.01, analysis not shown) with HLB-affected roots having a much lower lifespan than non-HLB controls, although differences were smaller at higher Cu treatments where lifespan was strongly reduced for the non-HLB controls (Figure 3).

**Figure 4:**
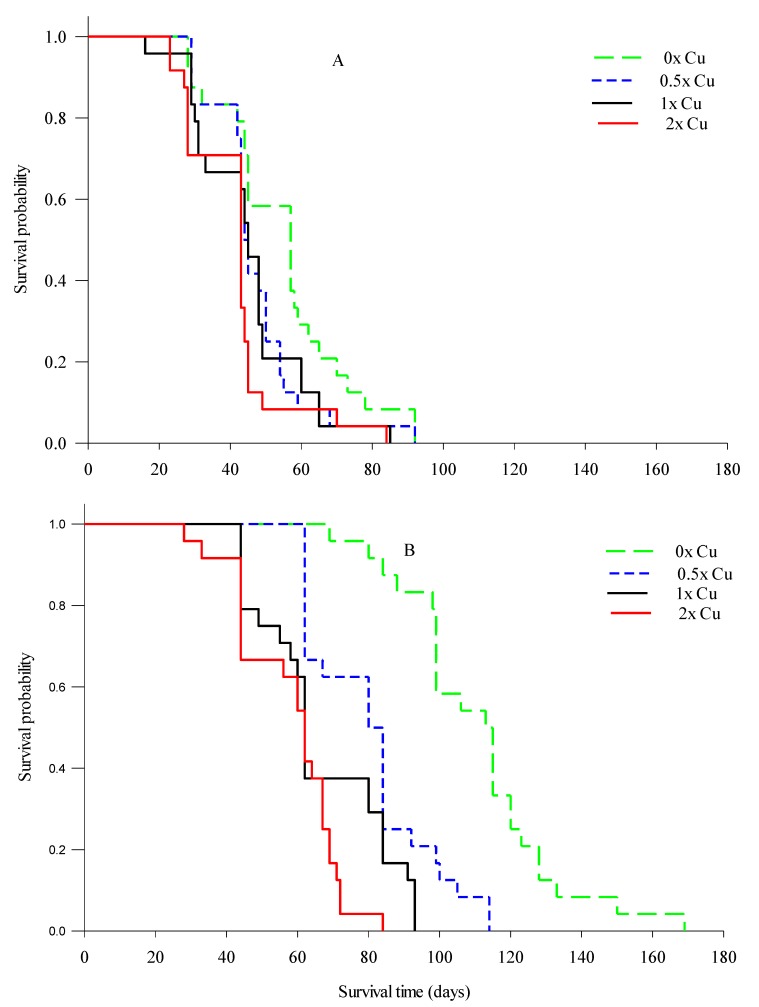
Interaction of HLB and foliar applications of Cu at 0×, 0.5×, 1×, and 2× the recommended rates (0, 46, 92, 184 mM, respectively) on root survival of HLB-affected (**A**) and healthy (**B**) citrus tree roots as demonstrated by Kaplan–Meier survival analysis models.

Half of the healthy roots observed survived more than 106 days, and three roots survived for 133 days (Table 2 and Figure 4). In contrast, all roots for HLB-affected trees lived less than 93 days with 50% roots lived less than 60 days. The maximum measured root lifespan was 169 and 92 days for healthy and HLB-affected trees, respectively. Generally, the average lifespan of HLB-affected trees represented 57% of the root lifespan of healthy trees. Similar observation was reported by Eissenstat and Yanai [21], who estimated that root lifespan for healthy citrus trees ‘Swingle citrumelo’ planted in Avon Park, Florida was 99 days. These results elucidate the impact of HLB on citrus root lifespan. As noticed earlier, increasing Cu rate to 2× significantly reduced the root lifespan of both HLB-affected and healthy trees by 36% and 24%, respectively.

### 3.4. T_sa_/U_sa_ (m^2^·m^−2^) 

Previous studies have demonstrated that HLB [2] and Cu treatments [46,47] suppress the growth of the root system. The current study has documented that the suppression of root growth corresponds to adjustment of the canopy such that the ratio of the transpiration surface area of the canopy to the uptake surface area of the root system (T_sa_/EU_sa_). Therefore, T_sa_/EU_sa_ was not affected by HLB or Cu treatment (Table 1). 

### 3.5. Ψ_xylem_ (MPa)

HLB caused a significant reduction in Ψ_xylem_, with the HLB-affected trees having an average Ψ_xylem_ of −1.07 MPa and the non-HLB controls having an average Ψ_xylem_ of −0.95 (Table 1), which was a 13% difference. Additionally, the time of sampling significantly (P <0.0001) affected tree Ψ_xylem_. A similar observation was reported in citrus trees under daily irrigation [7]. Unexpectedly, Cu treatments did not impact Ψ_xylem_ (P > F = 0.93). That could be a result of the balanced leaf area with tree roots development reduction.

**Figure 5:**
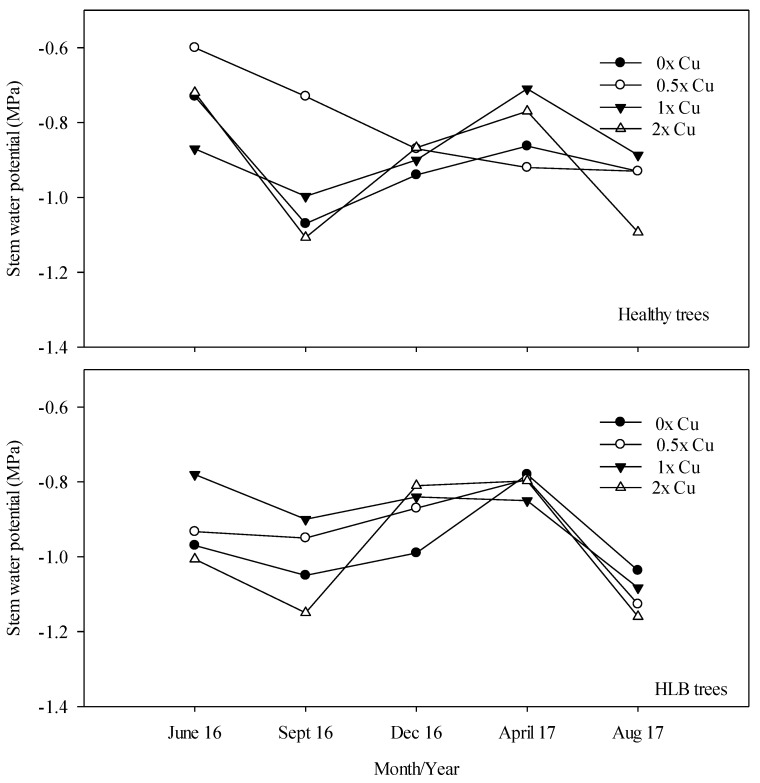
Interaction of HLB and foliar applications of Cu at 0×, 0.5×, 1×, and 2× the recommended rates (0, 46, 92, 184 mM, respectively) on stem water potential of *Citrus sinensis* cv. Valencia during 2016–2017.

The highest values of Ψ_xylem_ between −0.6 and −0.78 MPa (Figure 5) were observed during June 2016 under ‘Valencia’ 0× Cu healthy and HLB-affected trees, respectively. At the end of the experiment, these values decreased by 60% and 28% for HLB-affected and healthy trees, respectively. 

In general, lower Ψ_xylem_ were observed in September 2016 and August 2017 under 2× Cu rate with values between −1.2 and −1.11 MPa for HLB-affected and healthy trees, respectively. Similar results were observed under water-stressed crops under different environment [5,7,48,49]. 

### 3.6. Stomatal Conductance (k_s_)

The mildly lower Ψ_xylem_ for HLB-affected trees did not impact k_s_ (P > F = 0.82). HLB lowered trees’ k_s_ by only 2% more than that of healthy trees. Cu treatments did not impact Ψ_xylem_ (P > F = 0.93) or stomatal conductance (P > F = 0.10). However, results indicate that Cu applications represent a greater impact on stomatal aperture over time (P ˂ 0.01). Similar observations were reported by Gomes et al. and Syvertsen [49,50], who observed an increase in the rate of stomatal conductance for healthy citrus seedlings over time. 

Various researchers assumed that reductions in stomatal conductance values are indicators of water stress in citrus trees [51,52]. Syvertsen [53] reported that acute water stress (Ψ_xylem_ between −2.5 and −3.0) is responsible for the stomatal closure of orange trees. However, other researchers rejected that assumption and concluded other factors might be responsible for tree stress [54]. The current experiment demonstrates that water stress under irrigated citrus trees could be affected by tree health and common practices including Cu-containing materials.

### 3.7. T_p_ (mmoles·s^−1^)

There was a significant HLB*Cu*MAFT interaction (P > F < 0.01) in whole plant transpiration. In general, T_p_ increased over time similar to that of T_sa_, except in December where it was suppressed due to lower growth and less evaporative demand (figure not shown). Similarly, Espadafor et al. [55] observed a decline in the rate of transpiration for irrigated almond trees over time. On average, T_p_ was 2.5 mmoles·s^−1^ for HLB-affected trees and 3.0 mmoles·s^−1^ for the non-HLB controls, which represents a significant 17% difference. T_p_ also declined with increasing Cu rate applied from 3.8 mmoles·s^−1^ for the 0 mM treatment down to 2.1 mmoles·s^−1^ for the 184 mM application treatment, which represents an 80% difference. The reduction in T_p_ by HLB and increasing Cu rate is expected considering that these treatments produced smaller canopy areas. Our findings are consistent with those of other authors [47,56].

### 3.8. R_r+s_ (MPa·s·mmole^−1^)

R_r+s_ was not impacted by HLB or Cu treatment (Table 1). These results indicate that although HLB and Cu were suppressing root growth that had a corresponding impact on canopy growth, the effective ability of the root system to take up water was not impacted by either treatment. HLB-affected trees had lower R_r+s_ and increasing Cu rate to 2× increased root resistance by 16% compared with 0× Cu rate. Increased values from 2.1 × 10^−4^ to 2.5 × 10^−4^ MPa·s·mmole^−1^ were not enough to make R_r+s_ significantly different. It is generally accepted that R_r_ represents about 2/3 of the total resistance in the plant [4] and thus debilitation of the root system by stresses such HLB and/or Cu may lower R_r_, to which plants will respond by allowing Ψ_xylem_ to decline to some extent but compel stomates to close to prevent excessive plant water deficits. Changes in R_r+s_ were directly related to changes in water dynamics, including, Ψ_xylem_, stomatal conductance, transpiration, root surface area, and leaf area. Both Cu rate and HLB or separately, and some of their interactions significantly affected many of those measured parameters, as discussed separately. Previous studies indicated that citrus trees under a stressed environment might decrease root conductivity and increase root resistance and lower water uptake [24,57]. However, in the presented study, mild changes were reported as a result of both Cu rate and HLB. Even though the lifespan of HLB-affected and Cu-treated roots was significantly shortened, they were healthy functioning roots while alive.

## 4. Conclusions

HLB had a significant impact on plant water deficits compared to the healthy trees (13% difference), and Cu treatments did not impact plant water deficits, as measured by Ψ_xylem_. The levels of Ψ_xylem_ in this study indicated well-watered conditions, which typically do not influence k_s_ or impact growth [3,4,25]. However, HLB and Cu treatments impacted parameters that influence Ψ_xylem_ including T_sa_/U_sa_ and R_r+s_. HLB reduced T_sa_/U_sa_ by 30%, which would tend to favor water uptake and lessen plant water deficits, in addition, this benefit was combined with a lower R_r+s_ by 45%. The short lifespan of HLB-affected feeder roots and lower leaf area were likely the primary reasons for the lower R_r+s_ compared to the non-HLB controls. R_r+s_ increased with Cu treatment due to the strong impact on feeder roots, the total length of which declined by >3×. Root lifespan was also shortened by Cu treatment which decreases Rr. Stomatal conductance was not affected by HLB or Cu treatment which could be a result of growth impact reduction of HLB and Cu rates. Furthermore, T_sa_/U_sa_ was lowest for the 0 mM treatment where foliar Cu was in or near the sufficient range for most of the study. T_sa_/U_sa_ declined from a high of 18 for the 46 mM treatment down to 9 for the 184 mM treatment. These results indicate that the response of citrus trees exposed to Cu toxicity varies substantially from that of trees where foliar content of Cu is in or near the sufficiency range. Although, the results of this experiment indicate that HLB impacted some of the water relation parameters, and Cu application suppressed the growth of well-irrigated citrus trees, the plant water deficit was adjusted when the trees were affected by HLB or were treated with foliar applications of Cu, therefore, plant water deficits are not significantly impacted over those of the controls.

## Figures and Tables

**Table 1:** Impact of Huanglongbing (HLB) and foliar applications of Cu on foliar Cu concentration, vegetative growth, and water relations of *Citrus sinensis* cv. Valencia. Bolded P > F are referenced in the text where discussed. Acronyms are given in the footnotes ^1^.

Model Variables and Main Effects Means ^2^	Foliar Cu (mg·kg^−1^ Dry Weight)	T_sa_ (m^2^)	Total Observable Root Length (cm)		T_sa_/EU_sa_ (×10^4^ m^2^·m^−2^)	Ψ_xylem_ (MPa)	k_s_ (mmoles·m^−2^·s^−1^)	T_p_ (mmoles·s^−1^)	R_r+s_ (×10^−4^ MPa·s·mmole^−1^)
			Structural	Feeder					
HLB	0.01	0.01	0.31	<0.01	0.74	<0.01	0.82	<0.01	0.13
Cu	<0.01	0.30	0.03	<0.01	0.48	0.93	0.10	<0.01	0.75
HLB*Cu	<0.01	0.36	<0.01	0.02	0.50	0.94	0.83	0.01	0.71
MAFT	<0.01	0.09	0.15	0.47	0.20	<0.01	<0.01	<0.01	0.61
HLB*MAFT	<0.01	0.29	0.01	0.96	0.54	---	---	0.35	---
Cu*MAFT	<0.01	0.09	0.03	0.03	0.47	---	---	0.02	---
HLB*Cu*MAFT	<0.01	0.03	<0.01	<0.01	0.43	---	---	0.01	---
HLB treatment									
HLB	17.1a	2.1b	4.0	2.5b	8.3	−1.07a	180	2.5b	1.6
Control	12.0b	3.4a	7.7	5.3a	11.9	−0.95b	183	3.0a	2.9
Cu treatment (mM)									
0	4.9c	3.4a	9.5a	7.1a	3.9	−1.03	176	3.8a	2.1
46	13.6b	2.9ab	6.2b	4.5b	18.0	−0.98	194	3.1b	2.9
92	15.8b	2.5bc	4.3c	3.7bc	10.0	−1.01	182	2.4bc	1.8
184	23.7a	2.2c	3.4c	2.1c	9.0	−1.01	173	2.1c	2.5

^1^ T_sa_ = transpiration surface area of the canopy which is the same as leaf area, T_sa_/EU_sa_ is the ratio of leaf area to the estimated uptake surface area of the feeder roots, Ψ_xylem_ = xylem water potential of the stem, k_s_ = stomatal conductance, T_p_ = whole plant transpiration, and R_r+s_ = root and soil resistance. ^2^ HLB = Huanglongbing, Cu = foliar application of copper treatment, MAFT = months after first Cu treatment.

**Table 2:** Impact of HLB and foliar applications of Cu on foliar *Citrus sinensis* cv. Valencia root lifespan as described of the Kaplan–Meier estimate of the survival analysis during 2016 and 2017 measurements.

Time of Event-Day				Number of Roots Died				Live Roots at the Start of the Day (n)				Survival Probability				Standard Error			
Cu Rates–HLB Affected Roots																			
0×	0.5×	1×	2×	0×	0.5×	1×	2×	0×	0.5×	1×	2×	0×	0.5×	1×	2×	0×	0.5×	1×	2×
28	29	16	23	2	4	1	2	24	24	24	24	0.917	0.833	0.958	0.917	0.056	0.076	0.041	0.056
29	42	29	27	1	2	3	1	22	20	23	22	0.875	0.750	0.833	0.875	0.068	0.088	0.076	0.068
32	43	30	28	1	5	1	4	21	18	20	21	0.833	0.542	0.792	0.708	0.076	0.102	0.083	0.093
42	44	31	43	1	1	2	9	20	13	19	17	0.792	0.542	0.708	0.333	0.083	0.102	0.093	0.096
44	45	33	44	2	2	1	2	19	12	17	8	0.708	0.542	0.667	0.250	0.093	0.101	0.096	0.088
45	48	43	45	3	1	1	3	17	10	16	6	0.583	0.542	0.625	0.125	0.101	0.099	0.099	0.068
57	50	44	49	5	3	2	1	14	9	15	3	0.375	0.542	0.542	0.083	0.099	0.088	0.102	0.056
58	54	45	70	1	2	2	1	9	6	13	2	0.333	0.542	0.458	0.042	0.096	0.076	0.102	0.041
59	55	48	84	1	1	4	1	8	4	11	1	0.292	0.542	0.292	0.000	0.093	0.068	0.093	0.000
62	59	49		1	1	2		7	3	7		0.250	0.542	0.208		0.088	0.056	0.083	
65	68	60		1	1	2		6	2	5		0.208	0.542	0.125		0.083	0.041	0.068	
70	92	65		1	1	2		5	1	3		0.167	0.542	0.042		0.076	0.000	0.041	
73		85		1		1		4		1		0.125		0.000		0.068		0.000	
78				1				3				0.083				0.056			
92				2				2				0.000				0.000			
**Healthy Roots**																			
69	62	44	28	1	8	5	1	24	24	24	24	0.958	0.667	0.792	0.958	0.041	0.096	0.083	0.041
80	67	49	33	1	1	1	1	23	16	19	23	0.917	0.625	0.750	0.917	0.056	0.099	0.088	0.056
84	80	55	44	1	3	1	6	22	15	18	22	0.875	0.500	0.708	0.667	0.068	0.102	0.093	0.096
88	84	58	56	1	6	1	1	21	12	17	16	0.833	0.250	0.667	0.625	0.076	0.088	0.096	0.099
98	92	60	60	1	1	1	2	20	6	16	15	0.792	0.208	0.625	0.542	0.083	0.083	0.099	0.102
99	99	62	62	5	1	6	3	19	5	15	13	0.583	0.167	0.375	0.417	0.101	0.076	0.099	0.101
106	100	80	64	1	1	2	1	14	4	9	10	0.542	0.125	0.292	0.375	0.102	0.068	0.093	0.099
113	105	84	67	1	1	3	3	13	3	7	9	0.500	0.083	0.167	0.250	0.102	0.056	0.076	0.088
115	114	91	69	4	2	1	2	12	2	4	6	0.333	0.000	0.125	0.167	0.096	0.000	0.068	0.076
120		93	71	2		3	1	8		3	4	0.250		0.000	0.125	0.088		0.000	0.068
123			72	1			2	6			3	0.208			0.042	0.083			0.041
128			84	2			1	5			1	0.125			0.000	0.068			0.000
133				1				3				0.083				0.056			
150				1				2				0.042				0.041			
169				1				1				0.000				0.000			

## References

[1] Ebel R.C. (2017). Huanglongbing: Mechanism of Citrus Decline and Horticulture Management in Florida.

[2] Johnson E.G., Wu J., Bright D.B., Graham J.H. (2014). Association of ‘*Candidatus* Liberibacter asiaticus’ root infection, but not phloem plugging with root loss on Huanglongbing-affected trees prior to appearance of foliar symptoms. Plant Pathol..

[3] Kriedemann P.E., Barrs H.D., Kozlowsi T.T. (1981). Citrus orchards. Water Deficits and Plant Growth.

[4] Landsberg J.J., Jones H.G., Kozlowsi T.T. (1981). Apple orchards. Water Deficits and Plant Growth.

[5] Hamido S.A., Morgan K.T., Kadyampakeni D.M. (2017). The effect of Huanglongbing on young citrus tree water use. HortTechnology.

[6] Kumar N., Kiran F., Etxeberria E. (2018). Huanglongbing-induced anatomical changes in citrus fibrous root orders. HortScience.

[7] Hamido S.A., Morgan K.T., Ebel R.C., Kadyampakeni D.M. (2017). Improved irrigation management of sweet orange with Huanglongbing. HortScience.

[8] Driscoll P.J. (2004). Copper toxicity on Florida citrus—Why did it happen?. Proc. Fl. State Horticult. Soc..

[9] USDA-NASS (2019). Citrus Production by Type and Chemical Inventory by State. https://quickstats.nass.usda.gov/results/C6A49369-57C7-3F63-BE50-46FAE1A6B601.

[10] Bakshi S., He Z.L., Harris W.G. (2013). Particulate copper in soils and surface runoff from contaminated sandy soils under citrus production. Environ. Sci. Pollut. Res..

[11] Behlau F., Belasque J., Graham J.H., Leite R.P. (2010). Effect of frequency of copper applications on control of citrus canker and the yield of young bearing sweet orange trees. Crop Protect..

[12] Fan J., He Z., Ma L.Q., Stoffella P.J. (2011). Accumulation and availability of copper in citrus grove soils as affected by fungicide application. J. Soils Sediments.

[13] Ebel R.C., Hamido S., Morgan K.T. (2019). Interaction of Huanglongbing and foliar applications of copper on growth and nutrient acquisition of *Citrus sinensis* cv. Valencia. HortScience.

[14] Handique U., Ebel R.C., Morgan K.T. (2012). Influence of soil-applied fertilizer on greening development in new growth flushes of sweet orange. Proc. Fl. State Horticult. Soc..

[15] Li W., Hartung J.S., Levy L. (2006). Quantitative real-time PCR for detection and identification of *Candidatus Liberibacter* species associated with huanglongbing. J. Microbiol. Methods.

[16] Obreza T.A., Morgan K.T. (2008). Nutrition of Florida Citrus Trees, Cooperative Extension Service.

[17] Hanlon E.A., Gonzalez J.S., Bartos J.M. (1997). Institute of Food and Agricultural Sciences (IFAS) Extension Soil Testing Laboratory (ESTL) and Analytical Research Laboratory (ARL) Chemical Procedures and Training Manual.

[18] Jones J.B.J., Case V.W., Westerman R.L. (1990). Sample, Handling, and Analyzing Plant Tissue Samples.

[19] Plank C.O. (1992). Plant analysis reference procedures for the southern region of the United States. South Coop. Ser. Bull..

[20] Anderson D.L., Henderson L.J. (1988). Comparing sealed chamber digestion with other digestion methods used for plant-tissue analysis. Agron. J..

[21] Eissenstat D.M., Yanai R.D. (1997). The ecology of root lifespan. Advances in Ecological Research.

[22] Obreza T.A., Rouse R.E., Sherrod J.B. (1999). Economics of controlled-release fertilizer use on young citrus trees. J. Prod. Agric..

[23] Shabala S., White R.G., Djordjevic M.A., Ruan Y.L., Mathesius U. (2016). Root-to-shoot signaling: Integration of diverse molecules, pathways and functions. Funct. Plant Biol..

[24] Cohen Y. (1991). Determination of orchard water requirement by a combined trunk sap flow and meteorological approach. Irrig. Sci..

[25] Barkataky S., Morgan K.T., Ebel R.C. (2013). Water use of ‘Hamlin’ sweet orange during cold acclimation. Irrig. Sci..

[26] Ebel R.C., Proebsting E.L., Evans R.G. (2001). Apple tree and fruit responses to early termination of irrigation in a semi-arid environment. HortScience.

[27] Milliron L.K., Olivos A., Saa S., Sanden B.L., Shackel K.A. (2018). Dormant stem water potential responds to laboratory manipulation of hydration as well as contrasting rainfall field conditions in deciduous tree crops. Biosyst. Eng..

[28] Garnier E., Berger A. (1985). Testing water potential in peach trees as an indicator of water stress. J. Horticult. Sci..

[29] Alaoui-Sossé B., Genet P., Vinit-Dunand F., Toussaint M.L., Epron D., Badot P.M. (2004). Effect of copper on growth in cucumber plants (*Cucumis sativus*) and its relationships with carbohydrate accumulation and changes in ion contents. Plant Sci..

[30] Asati A., Pichhode M., Nikhil K. (2016). Effect of heavy metals on plants: An overview. Int. J. Appl. Innov. Eng. Manag..

[31] Cook C.M., Kostidou A., Vardaka E., Lanaras T. (1997). Effects of copper on the growth, photosynthesis and nutrient concentrations of Phaseolus plants. Photosynthetica.

[32] MacFarlane G.R., Burchett M.D. (2002). Toxicity, growth and accumulation relationships of copper, lead and zinc in the grey mangrove, Avicennia marina (Forsk.) Vierh. Mar. Environ. Res..

[33] Bevington K.B., Castle W.S. (1985). Annual root growth pattern of young citrus trees in relation to shoot growth, soil temperature, and soil water content. J. Am. Soc. Hortric. Sci..

[34] Marschner H. (1995). Mineral Nutrition of Higher Plants.

[35] Gao Y., Duan A., Qiu X., Liu Z., Sun J., Zhang J., Wang H. (2010). Distribution of roots and root length density in a maize/soybean strip intercropping system. Agric. Water Manag..

[36] Nickel S., Crookston R.K., Russelle M.P. (1995). Root growth and distribution are affected by corn–soybean cropping sequence. Agron. J..

[37] Abrisqueta J.M., Mounzer O., Álvarez S., Conejero W., García-Orellana Y., Tapia L.M., Veraa J., Abrisquetaa I., Ruiz-Sánchezab M.C. (2008). Root dynamics of peach trees submitted to partial rootzone drying continuous deficit irrigation. Agric. Water Manag..

[38] Bernier P.Y., Robitaille G. (2004). A plane intersect methods for estimating fine root productivity of trees from minirhizotrons images. Plant Soil.

[39] Comas L.H., Eissenstat D.M., Lakso A.N. (2000). Assessing root death and root system dynamics in a study of grape canopy pruning. New Phytol..

[40] Crocker T.L., Hendrick R.L., Ruess R.W., Pregitzer K.S., Burton A.J., Allen M.F., Shan J., Morris L.A. (2003). Substituting root numbers for length: Improving the use of minirhizotrons to study fine root dynamics. Appl. Soil Ecol..

[41] Ruiz-Sánchez M.C., Plana V., Ortuño M.F., Tapia L.M., Abrisqueta J.M. (2005). Spatial root distribution of apricot trees in different soil tillage practices. Plant Soil.

[42] Wells C.E., Glenn D.M., Eissenstat D.M. (2002). Changes in the risk of fine-root mortality with age: A case study in peach *Prunus persica* (Rosaceae). Am. J. Bot..

[43] Barney C.W. (1951). Effects of soil temperature and light intensity on root growth of loblolly pine seedlings. Plant Physiol..

[44] Reed J.F. (1939). Root and Shoot Growth of Shortleaf and Loblolly Pines in Relation to Certain Environmental Conditions.

[45] Wilcox H.E. (1962). Growth studies of the root of incense cedar, Librocedrus decurrens II. Morphological features of the growth system and root behaviour. Am. J. Bot..

[46] Zambrosi F.C.B., Mesquita G.L., Tanaka F.A.O., Quaggio J.A., Mattos D. (2013). Phosphorus availability and rootstock affect copper-induced damage to the root ultrastructure of *Citrus*. Env. Exp. Bot..

[47] Wutscher H.K., Smith P.F., Bennet W.F. (1996). Citrus. Nutrient Deficiencies & Toxicities of Crop Plants.

[48] Gasque M., Martí P., Granero B., González-Altozano P. (2016). Effects of long-term summer deficit irrigation on ‘Navelina’ citrus trees. Agric. Water Manag..

[49] Gomes M.D.M.D.A., Lagôa A.M.M.A., Medina C.L., Machado E.C., Machado M.A. (2004). Interactions between water potential, stomatal conductance and abscisic acid content of orange trees submitted to drought stress. Braz. J. Plant Physiol..

[50] Syvertsen J.P. (1994). Partial shoot removal increases net CO_2_ assimilation and alters water relations of Citrus seedlings. Tree Physiol..

[51] Davies F.S., Bower J. (1994). Water stress, gas exchange and fruit set of ‘Olinda’ Valencia orange trees in Eastern Transvaal area of South Africa. Acta. Horticult..

[52] Machado E.C., Medina C.L., Gomes M.D.M.D.A., Habermann G. (2002). Seasonal variation of photosynthetic rates, stomatal conductance and leaf water potential in ‘Valencia’ orange trees. Sci. Agricola.

[53] Syvertsen J.P. (1982). Minimum leaf water potential and stomatal closure in citrus leaves of different ages. Ann. Bot..

[54] Hamido S.A., Morgan K.T. (2018). Harvesting method affects water dynamics and yield of sweet orange with Huanglongbing. Agriculture.

[55] Espadafor M., Orgaz F., Testi L., Lorite I.J., González-Dugo V., Fereres E. (2017). Responses of transpiration and transpiration efficiency of almond trees to moderate water deficits. Sci. Horticult..

[56] Do Vale Gomes A.M.S., de Oliveira Reis F., de Lemos R.N.S., Mondego J.M., Braun H., Araujo J.R.G. (2019). Physiological characteristics of citrus plants infested with citrus blackfly. Rev. Bras. Entomol..

[57] Wilcox D.A., Davies F.S., Buchanan D.W. (1983). Root temperature, water relations, and cold hardiness in two citrus rootstocks. J. Am. Horticult. Sci..

